# Alternative SNP detection platforms, HRM and biosensors, for varietal identification in *Vitis vinifera* L. using *F3H* and *LDOX* genes

**DOI:** 10.1038/s41598-018-24158-9

**Published:** 2018-04-11

**Authors:** Sónia Gomes, Cláudia Castro, Sara Barrias, Leonor Pereira, Pedro Jorge, José R. Fernandes, Paula Martins-Lopes

**Affiliations:** 1 University of Trás-os-Montes and Alto Douro, P.O. Box 1013, 5000-911 Vila Real, Portugal; 2 University of Lisboa, Faculty of Sciences, BioISI – Biosystems & Integrative Sciences Institute, Campo Grande, Lisboa Portugal; 3 INESC TEC, Rua do Campo Alegre n. 687, 4169-007 Porto, Portugal; 4 CQVR and Department of Physics, University of Trás-os-Montes & Alto Douro, 5001-801 Vila Real, Portugal

## Abstract

The wine sector requires quick and reliable methods for *Vitis vinifera* L. varietal identification. The number of *V. vinifera* varieties is estimated in about 5,000 worldwide. Single Nucleotide Polymorphisms (SNPs) represent the most basic and abundant form of genetic sequence variation, being adequate for varietal discrimination. The aim of this work was to develop DNA-based assays suitable to detect SNP variation in *V. vinifera*, allowing varietal discrimination. Genotyping by sequencing allowed the detection of eleven SNPs on two genes of the anthocyanin pathway, the flavanone 3-hydroxylase (*F3H*, EC: 1.14.11.9), and the leucoanthocyanidin dioxygenase (*LDOX*, EC 1.14.11.19; synonym anthocyanidin synthase, *ANS*) in twenty *V. vinifera* varieties. Three High Resolution Melting (HRM) assays were designed based on the sequencing information, discriminating five of the 20 varieties: *Alicante Bouschet, Donzelinho Tinto, Merlot, Moscatel Galego and Tinta Roriz*. Sanger sequencing of the HRM assay products confirmed the HRM profiles. Three probes, with different lengths and sequences, were used as bio-recognition elements in an optical biosensor platform based on a long period grating (LPG) fiber optic sensor. The label free platform detected a difference of a single SNP using genomic DNA samples. The two different platforms were successfully applied for grapevine varietal identification.

## Introduction

*Vitis vinifera* L. is the world’s leading fruit crop mostly used in the wine industry. This is a highly adaptable species^1^ with a large number of varieties^2^. In Portugal, there are 343 varieties that can be used in wine production, wherein 240 are thought to be autochthonous^3^. The traditional methods for varietal identification and differentiation (ampleography and ampleometry) are based on morphological differences between the varieties. These methods present several restrictions, requiring alternative methods for variety identification^4^. The genes involved on the anthocyanins biosynthetic pathway, both structural and regulatory, have a direct effect in the anthocyanin profile of each variety and, therefore, are potentially interesting for varietal identification in the sense that the changes in their sequence may be linked to the differences found among the grapevine varieties^5^.

In 2007 the *V. vinifera* genome was completely sequenced^6,7^, allowing the identification of millions of genome-wide SNPs^8^. SNPs are changes in a single base at a specific position in the genome^9,10^. SNP markers present several advantages concerning varietal identification: mostly bi-allelic, highly abundant in genomes and a low mutation rate, which makes them stable during evolution^10,11^. Nowadays there are several methods for SNP identification^9,12^.

HRM is a simple, PCR-based method, for detecting DNA sequence variation by measuring changes in the melting of a DNA duplex^13^. HRM analysis can distinguish four classes of SNPs due to their different Temperature melting (Tm) shifts. SNP class 1 involves C/T and G/A and SNP class 2 involves C/A and G/T base exchanges that can easily be genotyped by HRM due to their high Tm differences of 0.5 °C. On the other hand, bases that only switch the strand are classified as SNP class 3 C/G base exchange and as SNP class 4 A/T base exchange, producing very small Tm differences of 0.4 °C and 0.2 °C, respectively^14^. HRM is highly sensitive and specific technique for SNP detection, providing a closed tube system, with a relatively low cost, with the exception of the equipment^15,16^.

Optical fiber LPGs, which can couple the core mode to the forward propagating cladding modes of a fiber, are widely used sensors to determine the fiber surrounding media refractive index (RI)^17^. LPGs transmission spectrum depends on the LPG period, length and local environment: temperature, axial strain, bend radius and on the RI of the medium surrounding the fiber^18^. The fiber optic biosensor based on a LPG used in this work has proven to be able to identify DNA hybridization events. This system is very sensitive and highly specific, allowing label free detection, without the need of any DNA amplification process^19^.

The aim of this study was to develop quick and reliable methods suitable for *V. vinifera* varietal discrimination, based on SNP information applied to HRM and biosensor platforms.

## Results and Discussion

### SNP detection in *F3H* and *LDOX* genes

The *F3H* is a single copy gene, responsible for the transcript of F3H protein located in a central position of the anthocyanin pathway. It has three exons and two introns and a length of 1,571 bp. The *LDOX* is a direct precursor enzyme of most of the flavonoid classes, anthocyanins. Previous reports show that the amount of anthocyanins is strongly related to the level of expression of *LDOX* gene^20^. *F3H* gene was sequenced in 20 grapevine varieties revealing the existence of ten SNPs (Table 1; Supplementary Figure S1), which translates in an average frequency of 1 SNP/157,1 nucleotides. The *LDOX* gene was also sequenced in the same grapevine varieties presenting a unique SNP in the nucleotide position 437 bp (Table 1). Both genes have a low SNP frequency when compared to a study by Lijavetzky and colleagues^21^ where a 100.5 Kb sequence, comprehending different gene fragments in 11 grape genotypes produced a higher frequency rate of 1 SNP/64 nucleotides. Also, Peukert and colleagues^22^ sequenced two nearby fragments of the *F3H* (1,339 bp) in 16 barley reference genotypes and obtained a frequency of 1 SNP/26.3 nucleotides.

**Table 1:** SNPs identified in F3H and in LDOX genes across the 20 grapevine varieties.

	Grapevine Code	Nucleotide position											
		4	47	291	1039	1040	1065	1157	1318	1381	1464	437	
	Reference	T	C	T	A	A	C	G	C	A	A	*C*	
***F3H***	**AB**	*	Y	*	W	W	*	*	Y	*	*	***LDOX***	*T*
	**CS**	G	*	*	*	*	*	C	*	G	T		*Y*
	**Ch**	K	*	*	*	*	*	S	*	R	W		*Y*
	**DT**	*	*	W	*	*	M	*	*	*	*		*
	**FP**	K	Y	*	W	W	*	S	Y	R	W		Y
	**Gou**	*	*	W	*	*	M	*	*	*	*		Y
	**MF**	*	Y	W	W	W	M	*	Y	*	*		*
	**M**	K	*	W	*	*	M	S	*	R	W		*
	**MG**	K	*	*	W	W	*	*	Y	*	*		Y
	**Ruf**	*	Y	*	W	W	*	*	Y	*	*		*
	**Sou**	G	*	*	*	*	*	C	*	G	T		Y
	**TA**	K	*	*	*	*	*	S	*	R	W		*
	**TB**	*	*	W	*	*	M	*	*	*	*		Y
	**TFi**	*	Y	W	W	W	M	*	Y	*	*		*
	**TR**	*	*	A	*	*	A	*	*	*	*		Y
	**TC**	G	*	*	*	*	*	S	*	R	W		*
	**TBr**	K	Y	*	W	W	*	S	Y	R	W		Y
	**TF**	K	*	*	*	*	*	S	*	R	W		Y
	**TN**	K	*	*	*	*	*	S	*	R	W		Y
	**Vio**	*	Y	*	W	W	*	*	Y	*	*		*

*Nucleotide Code*: A (Adenine); C (Cytosine); T (Thymine); G (Guanine); M (A or C); R (A or G); W (A or T); S (C or G); Y (C or T) and K (G or T).

*Equal to the described reference genotype.

Recently, Pereira and Martins-Lopes^5^ using the same set of grapevine varieties identified in UDP-Glucose:Flavonoid 3-O-Glucosyltransferase gene (*UFGT*) a large number of SNPs with a frequency of 1 SNP/25 nucleotides. This is a clear evidence that depending on the gene the SNP frequency varies largely within the same species. Moreover, the SNP frequencies varies within the same gene depending on the species.

The 10 SNPs discovered in the *F3H* gene are all bi-allelic, being seven transversions and three transitions, considering the reference genome. Three SNPs were found in exon 1, 3 SNPs in exon 3 with an average frequency of 1 SNP/128, and 1 SNP/115 nucleotides, respectively. Four SNPs were found in intron 2 with an average frequency of 1 SNP/51.8 nucleotides. The last SNP was found in the promoter region with an average frequency of 1 SNP/24 nucleotides. These results are in accordance with previous studies in barley^22^, grapevine^21^ and sunflower^23^ and are not surprising since exons account for 6.9% of the grapevine genome, and introns and intergenic sequences make up 36.7% and 34.7% of the genome, respectively^6^. The *F3H* sequence information differentiated four varieties of the 20 genotypes under study: *Merlot*, *Moscatel Galego*, *Tinta Roriz* and *Tinto Cão* (Table 1); however, it detected 10 different genotypes among the studied grapevine varieties. The *LDOX* gene sequencing information allowed the discrimination of one variety, the *Alicante Bouschet*, from the three detected genotypes (Table 1).

### HRM

The designed F3H HRM assays targeted two separated regions of the *F3H* gene: (1) a 375 bp fragment that includes two of the ten identified SNPs and; (2) a 532 bp fragment that includes seven SNPs (Supplementary Table S3).

The fragment length influences the sensitivity of the HRM analysis and literature refers that it shouldn’t be longer than 300 bp, resulting in rather complex melting profiles^24^. Despite this, Pereira and Martins-Lopes^5^ successfully used HRM in a 704 bp for varietal identification in *V. vinifera*, showing that HRM assays are still sensitive with long fragments.

Based on the different SNP patterns present in these two regions of *F3H* gene (Table 1) four and seven different haplotypes were detected in fragment F3H_375_bp_ and fragment F3H_532_bp_, respectively.

**Figure 1:**
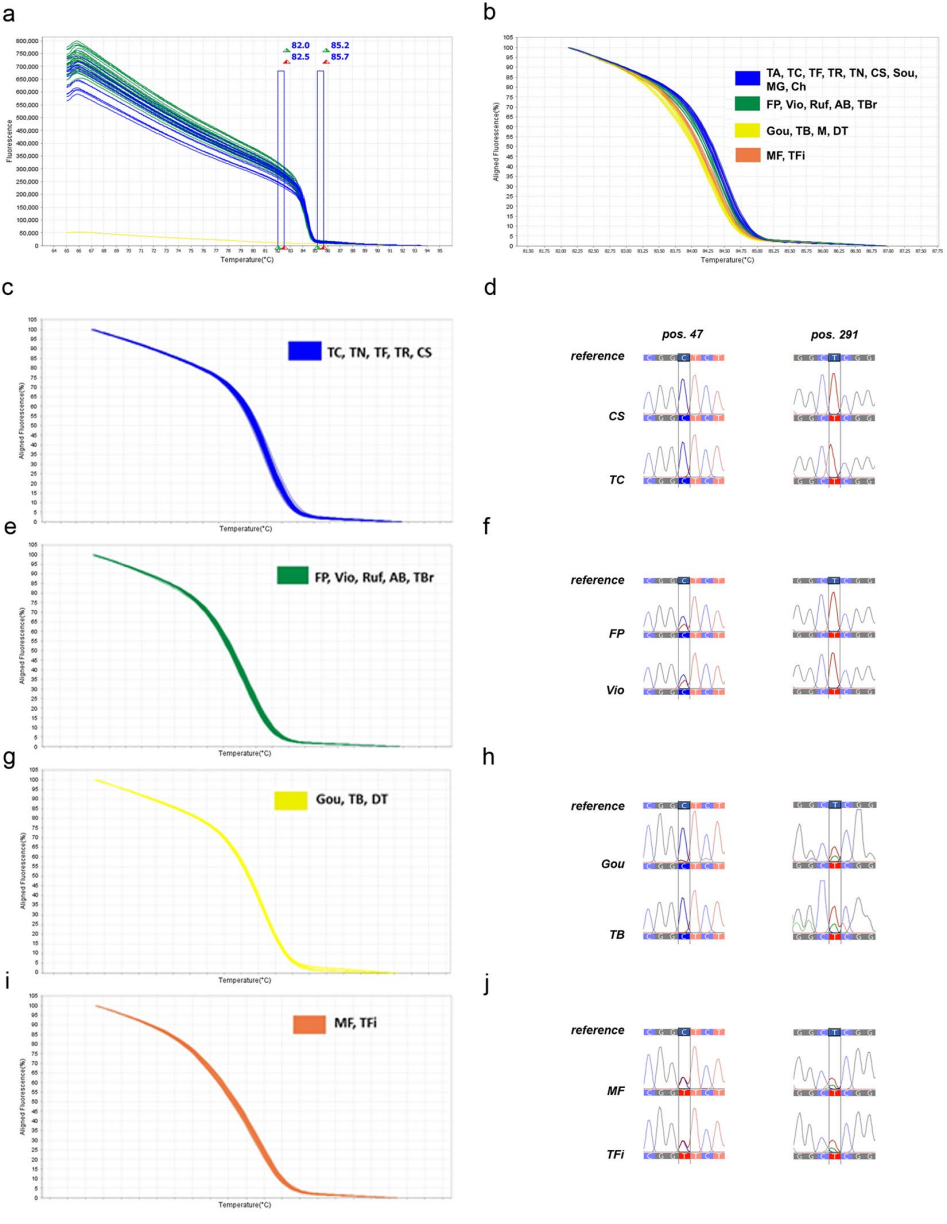
*F3H* gene HRM different profiles, based on T_m_, obtained for 375 _bp_ fragment. (**a**) Raw Data Melt Curve. Data collected during a HRM curve experiment showing pre and post-melt regions (double bars) used to align the data, producing a clearer view of the melt curve results. The curve colours represent 20 samples (AB - *Alicante Bouschet*; CS - *Cabernet Sauvignon*; Ch - *Chardonnay*; DT - *Donzelinho Tinto*; FP - *Fernão Pires*; Gou - *Gouveio*; MF - *Malvasia Fina*; M- *Merlot*; MG - *Moscatel Galego*; Ruf - *Rufete*; Sou - *Sousão*; TA - *Tinta Amarela*; TB - *Tinta Barroca*; TFi - *Tinta Francisca*; TR - *Tinta Roriz*; TC - *Tinto Cão*; TBr - *Touriga Brasileira*; TF - *Touriga Franca*; TN - *Touriga Nacional*; Vio - *Viosinho*). *O. europaea* L. spp. was used as negative control to obtain the raw data melt curve, no amplification occurred in this DNA sample. (**b**) General HRM profiles, based on T_m_, obtained for *F3H*_375_bp_ fragment revealing four different haplotypes. (**c–j**) Validation of *F3H*_375_bp_ different sequence variant using *V. vinifera* L. clones for each specific variety through Sanger sequencing. Each variety was validated using ten clones. (**c**,**d**) The same melting curve profiles was observed in clones from CS and TC, for nucleotide positions 47 and 291 bp, via melting curves normalization and sequencing HRM product. (**e**,**f**) HRM validation in FP and Vio varieties, shows the presence of variant Y [C or T] in nucleotide position 47 bp. (**g**,**h**) The presence of variant W [A or T] in nucleotide position 291 bp, in Gou and TB, was confirmed. (**i**,**j**) The evidence of SNPs in position 47 and 291 bp [C or T] was confirmed by sequencing the MF and TFi clones.

The F3H_375_bp_ HRM assay generated four melting curve profiles (Fig. 1a,b):

- Profile 1 clustered: *Tinta Amarela*, *Tinto Cão*, *Touriga Franca*, *Tinta Roriz*, *Touriga Nacional*, *Cabernet Sauvignon*, *Sousão*, *Moscatel Galego* and *Chardonnay*;
- Profile 2 clustered: *Fernão Pires*, *Viosinho*, *Rufete*, *Alicante Bouschet* and *Touriga Brasileira*;
- Profile 3 clustered: *Gouveio*, *Tinta Barroca, Merlot* and *Donzelinho Tinto*;
- Profile 4 clustered: *Malvasia Fina* and *Tinta Francisca*.

The different profiles can be distinguished by differences in T_m_ (Fig. 1b). The variety *Tinta Roriz* presents a unique homozygotic genotype (A/A) found at the position 291 bp (Table 1). However, TR was included in Profile 1 (homozygotic genotype T/T). The difference between *Tinta Roriz* and the remaining varieties consists in a class 4 SNP at the position 291 bp, where an A:T is present instead of an T:A. This difference between the sequences results in a very low T_m_ difference that is difficult to detect^14^.

**Figure 2:**
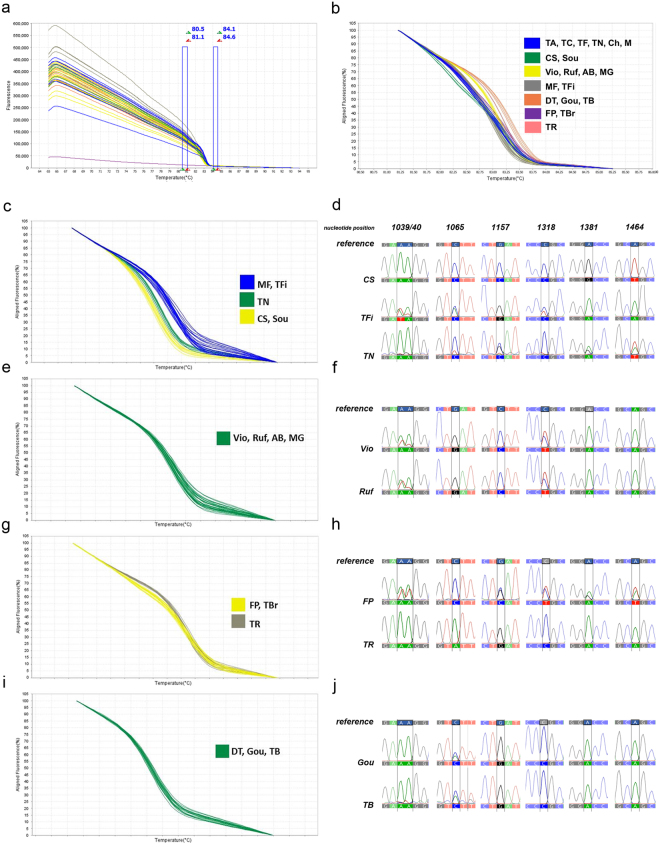
High-resolution DNA melting curve analysis for a 532 _bp_ amplicon with seven haplotypes in *F3H* gene. (**a**) Raw Data Melt Curve. Data collected during a HRM curve experiment showing pre and post-melt regions (vertical bars) that are used to align the data, producing a clearer view of the melt curve results. A melting curve generated by a DNA negative control (*O. europaea* L.) was used, no amplification was observed. The curve colours represent the sequence variations among 20 samples (AB - *Alicante Bouschet*; CS - *Cabernet Sauvignon*; Ch - *Chardonnay*; DT - *Donzelinho Tinto*; FP - *Fernão Pires*; Gou - *Gouveio*; MF - *Malvasia Fina*; M - *Merlot*; MG - *Moscatel Galego*; Ruf - *Rufete*; Sou - *Sousão*; TA - *Tinta Amarela*; TB - *Tinta Barroca*; TFi - *Tinta Francisca*; TR - *Tinta Roriz*; TC - *Tinto Cão*; TBr - *Touriga Brasileira*; TF - *Touriga Franca*; TN - *Touriga Nacional*; Vio - *Viosinho*). (**b**) HRM profiles, based on *F3H*_532_bp_ fragment T_m_, revealing seven haplotypes. (**c–j**) Validation of *F3H*_532_bp_ sequence variants by Sanger sequencing using *V. vinifera* L. clones. Ten clones of each variety were used to validate HRM assay with the coincident melting curve profiles. (**c**,**d**) HRM product validation in CS, TFi and TN varieties, display the presence of variant in position 1039/40 [A or T], 1065 [A or C], and 1318 bp [C or T] for TFi variety. The sequence validation for TN was confirmed by SNPs presence in positions 1157 [C or G], 1381 [A or G], and 1464 bp [A or T]. (**e**,**f**) The evidence of SNPs in position 1039/40 [A or T], and 1318 bp [C or T] was confirmed by sequencing Vio and Ruf clones. (**g**,**h**) FP clones validate the variations of HRM assay for nucleotide position 1039/40 [A or T], 1157 [C or G], 1318 [C or T], 1381 [A or G] and 1464 bp [A or T]; and TR clones for position 1065 bp [A]. (**i**,**j**) The evidence of SNPs [A or C] in position 1065 bp was confirmed by sequencing Gou and TB clones.

The F3H_532_bp_ HRM assay generated seven melting curve profiles, as predicted (Fig. 2a,b):

- Profile 1 clustered: *Tinta Amarela*, *Tinto Cão*, *Touriga Franca*, *Touriga Nacional, Merlot* and *Chardonnay*;
- Profile 2 clustered: *Cabernet Sauvignon* and *Sousão*;
- Profile 3 clustered: *Viosinho*, *Rufete*, *Alicante Bouschet* and *Moscatel Galego*;
- Profile 4 clustered: *Malvasia Fina* and *Tinta Francisca*;
- Profile 5 clustered: *Donzelinho Tinto*, *Gouveio* and *Tinta Barroca*;
- Profile 6 clustered: *Fernão Pires* and *Touriga Brasileira*;
- Profile 7 is specific of the variety *Tinta Roriz*.

In F3H_532_bp_ the melting profiles are based on different curves shapes (Fig. 2b), unlike F3H_375_bp_. These type of melting curves are characteristic of fragments that melt in two different domains.

*Tinta Roriz* has a unique melting profile in fragment F3H_532_bp_ with a nucleotide variation in position 1065 bp, presenting a homozygotic genotype (A/A). In this HRM assay *Tinta Roriz* was clustered independently, as a result of the SNP combination present in this gene region. These results confirmed the sensitivity and specificity of HRM technique.

The LDOX HRM assay targeted one region of the gene that include the unique SNP identified within the sequence (450 bp fragment; Supplementary Table S3).

**Figure 3:**
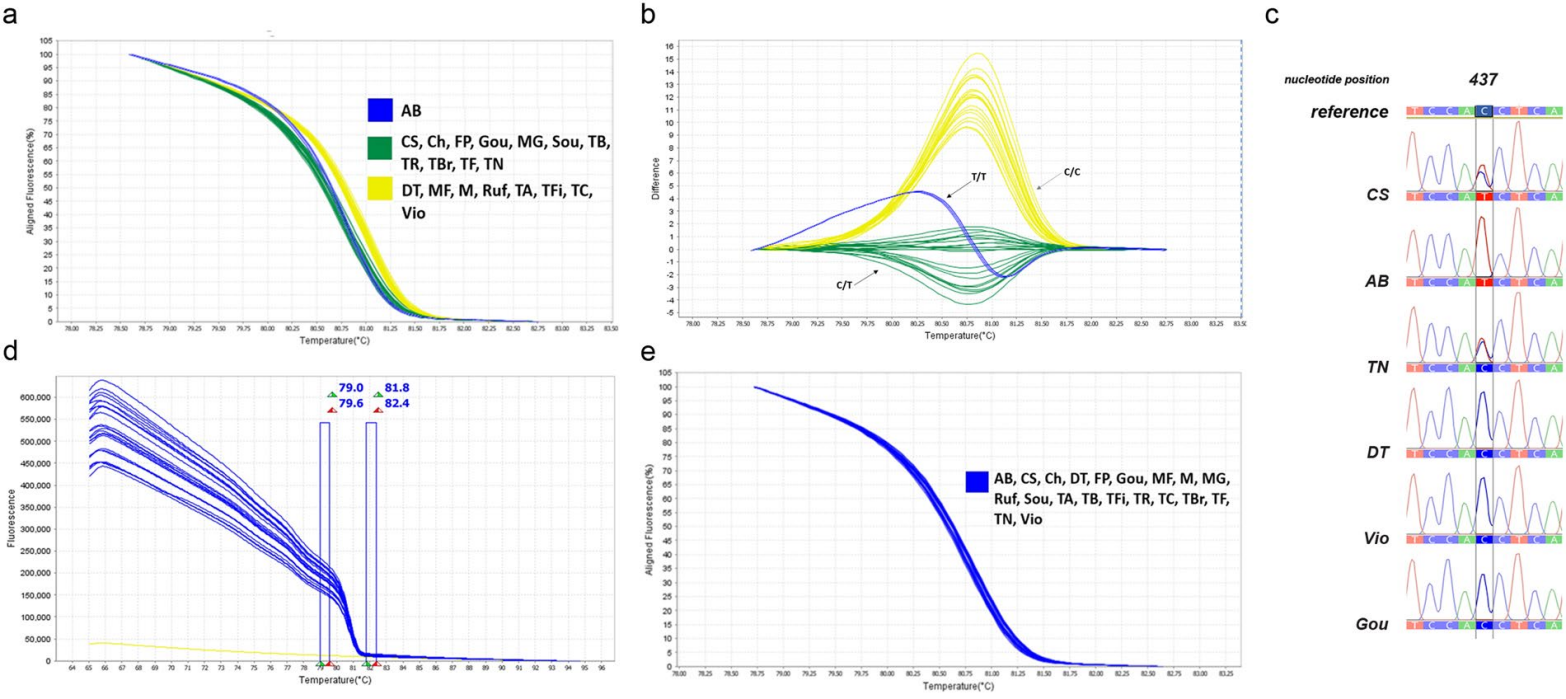
HRM analysis of grapevine varieties for the *LDOX*_450_bp_ fragment. (**a**) Aligned melting curves, and (**b**) Difference plot correspond to two representations of the same data obtained in LDOX_450_bp_ HRM assay, using 20 varieties, and revealing three distinct genotypes. (**c**) Validation of *LDOX_450*_bp_ SNP [T/T], [C/C] and [C or T] in nucleotide position 437 bp, by Sanger sequencing. Different grapevine varieties formed groups of melting curves, according their sequence variations. Three haplotypes are detected through HRM assay: homozygote SNP [**C/C** : *DT, MF, M, Ruf, TA, TFi, TC, Vio*], heterozygote [**C or T** : *CS, Ch, FP, Gou, MG, Sou, TB, TR, TBr, TF, TN*], and homozygote SNP [**T/T** : *AB*]. (**d**) Raw Data Melt Curve based on T_m_, obtained for 201 _bp_ fragment. Data collected during HRM curve experiment showing pre- and post-melting normalization regions (double columns) that are used to align the data, producing a clearer view of the melt curve results. *O. europaea* L. spp. was ussed as negative control, no amplification occurred. (**e**) The specificity of the HRM as technique was shown by a single melt curve (blue) using the 20 varieties (*AB*; *CS*; *Ch*; *DT*; *FP*; *Gou*; *MF*; *M*; *MG*; *Ruf*; *Sou*; *TA*; *TB*; *TFi*; *TR*; *TC*; *TBr*; *TF*; *TN*; *Vio*) for *LDOX*_201_bp_ fragment amplification, and where no sequence variation was observed.

In the LDOX_450_bp_ HRM assay samples were grouped based on the melting curve shape showed by the normalized melting curves (Fig. 3a) and difference plot (Fig. 3b). Thus, LDOX_450_bp_ generated three melting curve profiles:

- Profile 1 is specific of the variety *Alicante Bouschet;*
- Profile 2 clustered: *Cabernet Sauvignon*, *Chardonnay*, *Fernão Pires*, *Gouveio*, *Moscatel Galego*, *Sousão*, *Tinta Barroca*, *Tinta Roriz*, *Touriga Brasileira*, *Touriga Franca* and *Touriga Nacional*;
- Profile 3 clustered: *Donzelinho Tinto, Malvasia Fina, Merlot, Rufete, Tinta Amarela, Tinta Francisca, Tinto Cão*, and *Viosinho*.

The *Alicante Bouschet* clustered independently based on its unique haplotype, homozygous (T/T) (Fig. 3a,b).

The feasibility of HRM technique was validated by an assay designed in a specific *LDOX* gene region without any sequence variation, among the twenty varieties under study (Fig. 3d,e). Additionally, no amplification occurred in DNA samples of non-*V. vinifera* L. varieties, and thus no melting curve was observed, showing its specificity (Figs 1a, 2a and 3a).

Based on the melting curve profiles three grapevine variety were individually distinguished: 1) *Moscatel Galego* presenting a unique profile combination (F3H_375_bp_ Profile 1, and F3H_532_bp_ Profile 3, and LDOX_450_bp_ Profile 2); 2) *Donzelinho Tinto* (F3H_375_bp_ Profile 3, F3H_532_bp_ Profile 5, and LDOX_450_bp_ Profile 3) and; 3) Merlot (F3H_375_bp_ Profile 3, F3H_532_bp_ Profile 1, and LDOX_450_bp_ Profile 3).

Grapevine clones of the same variety behave differentially and present some genetic differences among them^25,26^. Therefore, when a specific genetic marker is developed for varietal identification there is a need to validate it in the existing clones. Clones of each grapevine variety were used to validate the HRM profiles using the same assay conditions. The different profiles presented are consistently reproduced between samples and the respective clones (Figs 1c–j and 2c–j).

Other techniques have been used to discriminate grapevine varieties. Tessier and colleagues^27^ were able to discriminate 224 varieties with the combination of 6 RAPDs (Random Amplified Polymorphic DNA) and 2 SSRs markers (Simple Sequence Repeats). Also, Faria and colleagues^28^ used 4 SSRs to discriminate only 5 varieties. More recently, Castro and colleagues^29^ used 12 SSRs to discriminate 39 varieties and Pereira and colleagues^30^ used 6 SSRs to discriminate 11 varieties. Although the studies mentioned discriminated more varieties, they analysed a higher number of genome regions than the present study. The analysis of more polymorphic regions will increase the probability of a wider varietal differentiation^5^. Such could be accomplished by obtaining unique profiles or by combining profiles from different genome regions. Based on both *F3H* HRM assays nine genotypes were identified among the twenty varieties under study wherein three are unique: *Merlot*, *Moscatel Galego* and *Tinta Roriz*. The HRM *LDOX* assay allowed the discrimination of three genotypes, where the *Alicante Bouschet* variety showed a unique profile. Combining the three HRM assays a total of twelve genotypes were found, wherein, five are unique: *Alicante Bouschet*, *Donzelinho Tinto*, *Merlot*, *Moscatel Galego* and *Tinta Roriz*. Furthermore, the development of HRM assays capable of differentiating more varieties can allow a faster and cost-effective manner to analyse a larger number of samples since several SNPs can be analysed simultaneous, without the use of time consuming detection methods, such as agarose gel or capillary electrophoresis, as other molecular marker systems requir^5^.

### Biosensor assay

The LPG biosensor was developed within the group as a new method for SNP detection. Two different size probes were used. Probe 25 with a sequence of the *F3H* gene (NCBI reference) comprehending the 1,026 to the 1,050 bp region (Table 1). Probe 35 and 1065 is positioned in the same *F3H* gene from 1,035 to 1,069 bp. In order to be certain that the Probes designed for *F3H* gene were specific a blast was performed against the *V. vinifera* genome and all data available in the NCBI database.

**Figure 4:**
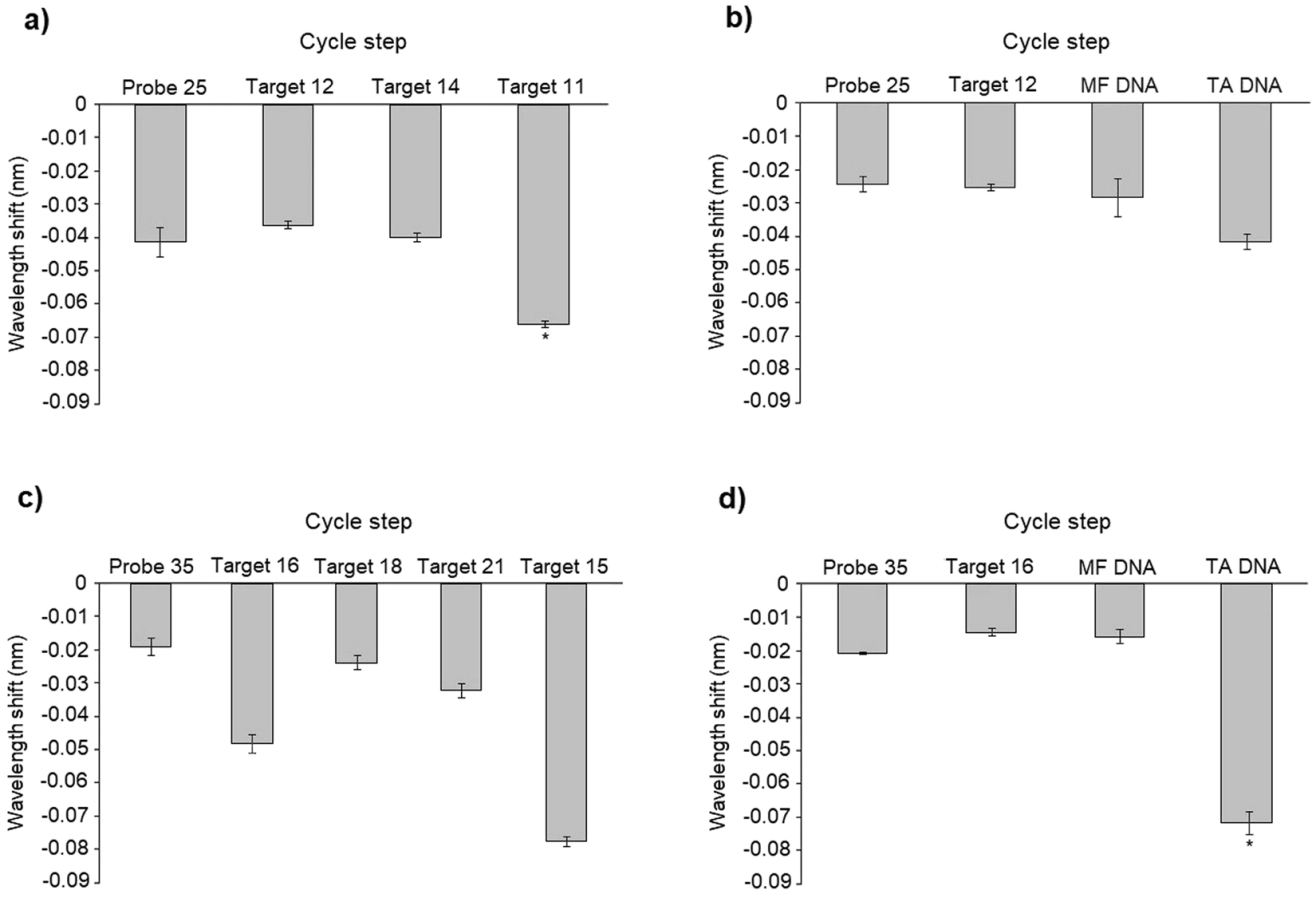
Representation of the sensor response in the presence of: (**a**) single strand DNA-Probe 25, Target 12 (single mismatch), Target 14 (two mismatches), and Target 11 (complementary); (**b**) single strand DNA-Probe 25, Target 12 (single mismatch), MF DNA (two mismatches), and TA DNA (complementary). (**c**) single strand DNA-Probe 35, Target 16 (single mismatch), Target 18 (two mismatches), Target 21 (three mismatches), and Target 15 (complementary); (**d**) single strand DNA-Probe 35, Target 16 (single mismatch), MF DNA (three mismatches), and TA DNA (complementary). *The values are statistically significant when compared to the Probe Signal for a P < 0.05.

Probe 25 was tested with Targets 11, 12 and 14 (Fig. 4a). Targets 12 and 14 (with one and two SNPs, respectively) presented a similar wavelength shift to Probe 25, indicating that no hybridization occurred. On the other hand, Target 11 (complementary sequence), clearly hybridized, having almost a 60% signal increase when compared to Probe 25 signal (Fig. 4a).

The use of real DNA samples is the ultimate test, once the biosensor is targeted for real life application. Probe 25 was tested with *Malvasia Fina* (MF; two mismatches) and *Tinta Amarela* (TA; complementary) leaf DNA (Fig. 4b). Target 12 was used as a negative control. Target 12 and MF DNA presented similar wavelength shifts to Probe 25, meaning that no hybridization occurred (Fig. 4b). When TA DNA was used there is a significant (P < 0.05) signal increase in comparison to Probe 25, with a 70% difference, meaning that hybridization occurred (Fig. 4b). The sensor was able to correctly identify a real DNA sample, which is considerably more complex than the targets, without any signal loss. In addition, the fact that there was no hybridization with the MF DNA proves that probe 25 is specific to a unique sequence in the *V. vinifera* genome, and that this sequence is suitable to distinguish between these two varieties.

Probe 35 was tested with Targets 15, 16, 18 and 21 (Fig. 4c). The signals obtained with Targets 16, 18 and 21 were higher than expected, however all of them were still expressively lower than the signal increase of Target 15, which presented a significant (P < 0.05) signal increase of 300% when compared to Probe 35. Considering this, the signals obtained for the targets without a fully complementary sequence are still considered negative.

Probe 35 was tested with Target 16, MF DNA and TA DNA (Fig. 4d). In this experiment, Target 16 was the negative control, presenting a similar wavelength shift to MF DNA and the Probe 35, indicating that no hybridization occurred. A significant (P < 0.05) signal increase was verified when TA DNA was added, being 240% higher than the Probe 35 signal, indicating that hybridization occurred.

When comparing the signal difference between Probe 35 and Target 16 in the two experiences it is evident that some abnormally occurred in the first experience, however no certain explanation can be advanced in this case. Although user error cannot be ruled out, it is unlikely because each experience was repeated three times and they all had a similar behaviour. Other possible explanation is undetected temperature/tension shifts, since the LPGs are very sensitive to this two parameters^19^. The results obtained with real DNA samples are very promising since there was just a small signal loss, ca. 8%, when TA DNA is compared to Target 15, which is quite amazing once there are competitive sequences within the sample. Once again, the lack of hybridization of the MF DNA proves the specificity of the designed probe.

The sensor was able to detect a difference of only one SNP with both probes, proving that this system is very specific and sensitive as was reported by Gonçalves and colleagues^19^. As far as our knowledge, this is the first time that such a long probe (35 bases) was tested in this sensor type. By the results obtained it seems that a probe length increase leads to a signal increase in the correctly hybridized samples, since probe 25 only had a ca. 60% signal increase with the complementary Target 11, while Probe 35 presented a ca. 300% increase when using Target 15. On the other hand, when real DNA samples were used, with Probe 25 there was zero signal lost, whereas when Probe 35 was used there was a small decrease in the signal, which may indicate that probe size can improve the signal until a certain limit when samples are more complex.

**Figure 5:**
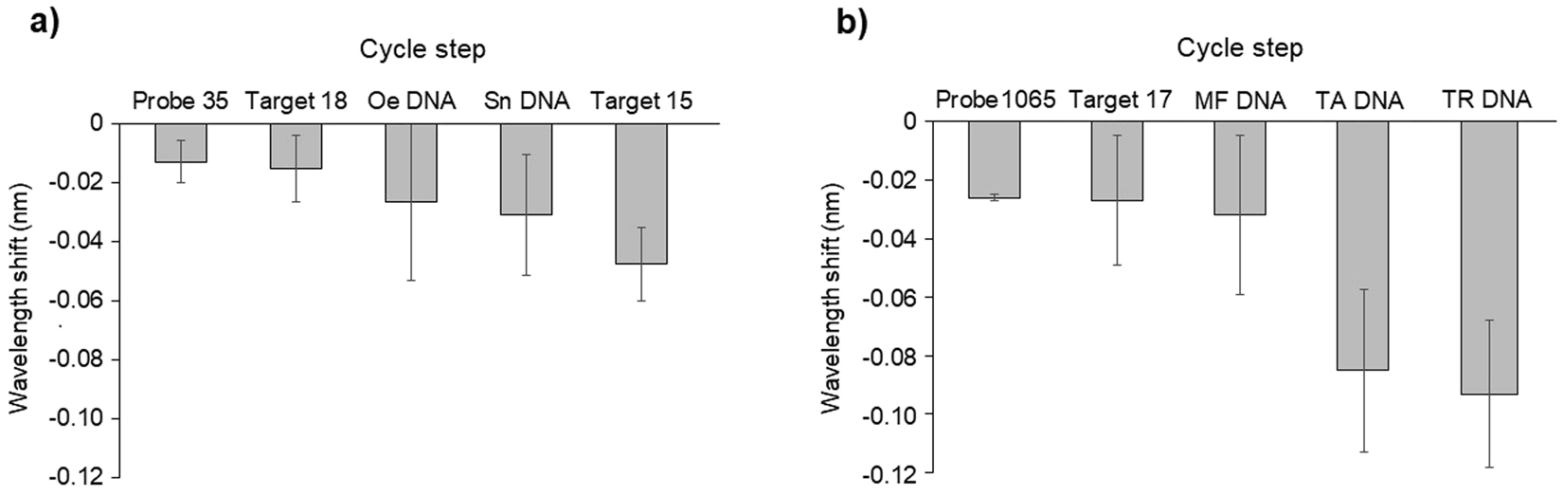
Representation of the sensor response in the presence of: (**a**) single strand DNA-Probe 35, Target 18 (two mismatches), gDNA of *O. europaea* (negative), gDNA of *S. nigra* (negative), and Target 15 (complementary); (**b**) single strand DNA-Probe 1065 (length 35 bases specific for grapevine variety TR- *Tinta Roriz*), Target 17 (two mismatches), gDNA MF-*Malvasia Fina* (position: 1039 bp – A/T; 1040 bp – A/T; 1065 bp – A/C); gDNA TA- *Tinta Amarela* (position: 1039 bp – A; 1040 bp – A; 1065 bp - C); and gDNA TR – *Tinta Roriz* (position: 1039 bp – A; 1040 bp – A; 1065 bp – A).

Even though the probes had been checked for specificity through a NCBI blast, the use of different species DNA (Oe - *Olea europaea* L. and Sn - *Sambucus nigra* L.) were tested (Fig. 5a). The signal increased slightly with the DNA from the Oe and Sn sources, nevertheless the signal increase was within the limit observed previously with non-complementary targets (Fig. 4). When the complementary sequence, Target 15, was added the signal increase in relation to the probe is significantly higher, proving the specificity of the designed probe.

**Figure 6:**
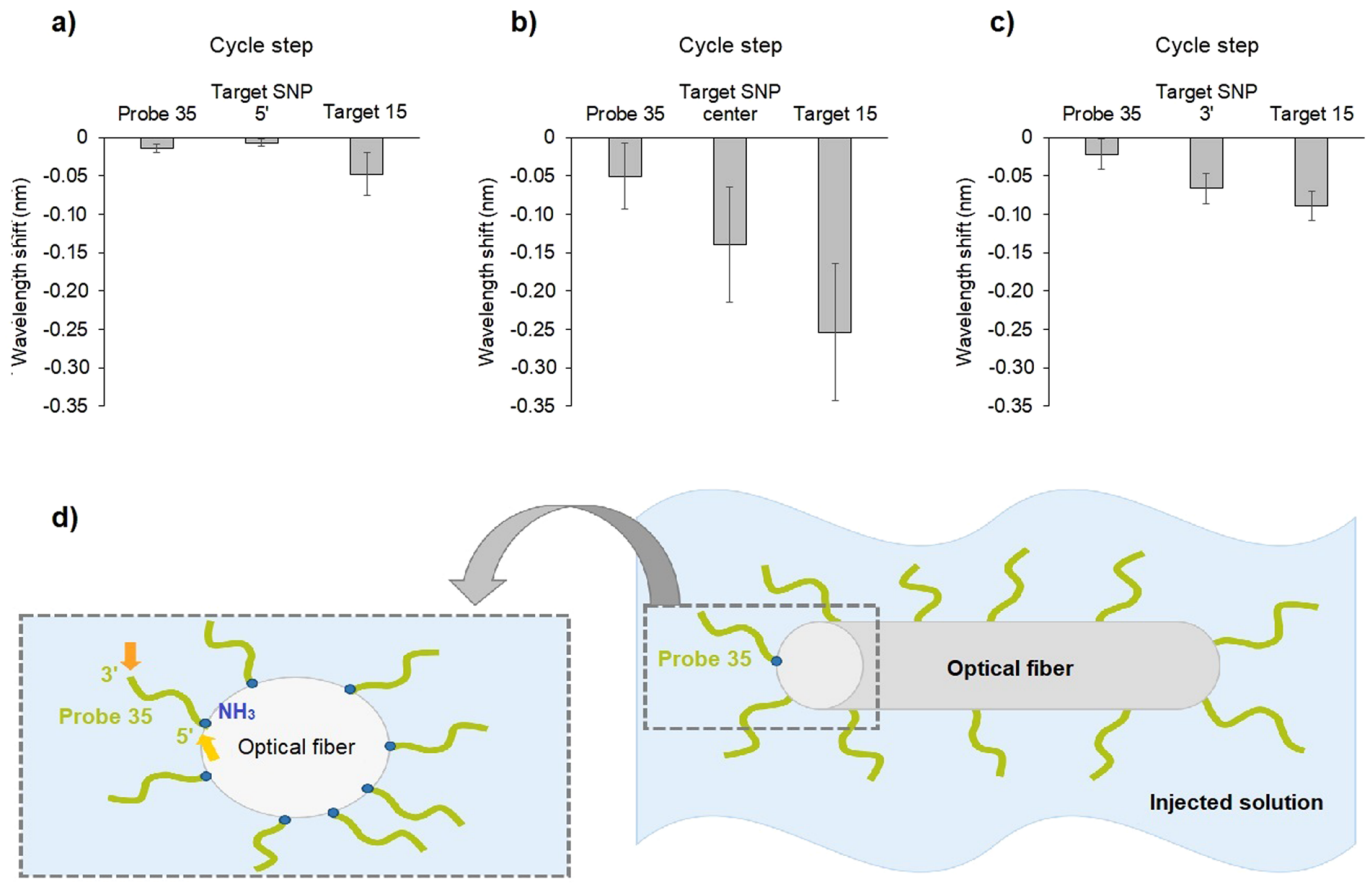
Representation of the sensor response with a SNP positioned in different locations in relation to the probe: (**a**) single strand DNA-Probe 35, Target SNP 5′ (one mismatch in the 5′ end of the target), and Target 15 (complementary); (**b**) single strand DNA-Probe 35, Target SNP center (one mismatch in the center of the target), and Target 15 (complementary); (**c**) single strand DNA-Probe 35, Target SNP 3′ (one mismatch in the 3′ end of the target), and Target 15 (complementary); (**d**) representation of the probe immobilization process on the fiber, with a detailed vision of the top of the fiber and the disposition of the probe, with a wider access in the 3′-end of the probe (orange arrow) favouring the beginning of the hybridization reaction with the samples that are present in the fiber’s surrounding environment.

A new probe was included, specific of TR, with exactly the same length as Probe 35, only deferring at the position 1065 (Supplementary Table S4). This Probe provided the expected results for Target 17 and gDNA from MF, not presenting hybridization. However, when gDNA from TA was used the signal increased significantly, coming to a similar level as detected for TR (correspondent sample). The TA hybridized with the TR specific probe, as Probe 1065 included the positions 1039 and 1040 (Table 1) which are identical for both grapevine varieties. Whereas MF is differentiated at these two positions, therefore not hybridizing with the probe. This experience and the previous results obtained with target 16 (Fig. 4c) rose questions relatively to the specificity of the probe, leading to another set of experiences to better understand the hybridization process in this platform. Thus, a set of three targets were designed complementary to Probe 35 except for a unique SNP that varied in its position in the target at the: 5′-end; centre and, 3′-end (Fig. 6a,b,c, respectively). The hybridization was influenced by the SNP position in the target, being clear that if the SNP is positioned at the 5′-end of the target sequence the hybridization does not take place (Fig. 6a); whereas, if the SNP is in a centre position the reaction is variable (sometimes it hybridizes, sometimes it doesn’t) and; when it is in 3′-end of the target it always hybridizes. Thus, the position of the SNP when designing probes should be in the 3′-end to guarantee that no mismatches will occur, once it is there were preferentially the targets will start to hybridize once it is farer from the fiber (Fig. 6d).

As all the experiments were conducted using the same molarity, 0.25 µM, the number of estimated copies between the synthetic targets and the real sample are considerably different, being 5.35 E^+^ ^16^ for Target 11, 7.599 E^+^ ^16^ for Target 15 and 2.687 E^+^ ^6^ for the real sample. This emphasis the sensibility of the method when using direct real DNA samples, and the fact that no previous amplification step was required, increasing the target number in the solution as is required for some actually used DNA-based biosensors^31^. This, is an interesting feature as some DNA samples present PCR-inhibitors^32^, limiting their use for genotyping procedures.

### HRM vs LPG biosensor

The development of quicker, cheaper and reliable SNP detection methods are a hot topic in DNA technology^10^. In this study, two recent technologies have been tested. HRM analysis uses real-time PCR instrumentation to analyse DNA sequence variation. Biosensor technology is performed by exploiting the interaction of the optical field with a biorecognition element. Both technologies were successful used in SNP detection, presenting advantages and disadvantages.

HRM requires a previous PCR amplification step, to increase the number of target DNA molecules containing SNPs, whereas a DNA-based biosensor does not require an amplification step. When considering time request, HRM is considerably faster. The HRM protocol itself is single-step, closed-tube method and allows multiple sample analysis in one single assay. In the biosensor assay, the temperature stabilization is time consuming and increases substantially the time required for data acquisition in each step. More even, the setup is still limited allowing the analysis of a single sample. The biosensor can only detect a single genotype at a time for each specific probe, whereas HRM can detect multiple genotypes as long as the primer site does not present a mutation and there are several differentiating SNPs. More even, the biosensor does not require a specialized user and the data interpretation is simpler, when compared to HRM. Furthermore, it is cheaper, because the probe can be reused and no fluorescent dyes nor enzymes are required.

Although the biosensor system still presents some disadvantages in relation to the HRM, the two technologies are at different development levels.

### Concluding remarks

The demand for cheap, reliable and quick SNP detection methods are a highlight topic. HRM assays and a DNA-based biosensor were designed for grapevine varietal identification, based on the SNPs identified in the *F3H* and *LDOX* genes. The described HRM assays fulfilled our objectives and permitted an accurate identification of the targeted varieties, allowing their extended application for other research purposes, such as grapevine germplasm screening.

DNA-based biosensors are an expanding technology that is being applied in a large number of sectors. Still, the DNA-based biosensor needs further mechanical and electronic improvement in order to be suitable for multiple event detection in a shorter time period.

In the future, with the advances in high-throughput sequencing platforms, it is foreseen that more SNPs will be identified, which will allow the discrimination of all grapevine varieties. Together, with the development of alternative SNP detection platforms, such as the ones herein described, it will be possible to control the grapevine varieties throughout the grape-wine chain.

## Methods

### *F3H* sequencing and HRM assays

Twenty grapevine varieties were studied (Supplementary Table S1). Young leaves of each grapevine variety and of their clones were collected, and immediately frozen in liquid nitrogen, from the Sogrape Vinhos S.A., Real Companhia Velha, Sociedade Borges S.A., the Direção Regional de Agricultura e Pescas do Norte and to the National Ampelographic Collection (Dois Portos).

Total genomic DNA was extracted from the frozen young leaves using the CTAB method^33^. The purity, integrity and quantity of all DNA samples was estimated by Nanodrop™ 1000 Spectrophotometer (Thermo Fisher Scientific) measurements and by electrophoresis on a 0.8% agarose in 1 × TAE (Tris-acetate-EDTA).

Amplicon resequencing strategy was used to detect SNPs in the *F3H* and *LDOX* genes in the different varieties. These genes were amplified using the primers listed in Supplementary Table S2. Primers were designed in the Primer3Plus software^34^ based on the *F3H* and *LDOX* sequence available in NCBI (http://www.ncbi.nlm.nih.gov/gene/?term=F3H+-vitis-+vinifera, date of access: 23-04-2015; and https://www.ncbi.nlm.nih.gov/gene/?term=LDOX+-vitis-+vinifera, date of access: 20-11-2016). The PCR reactions were performed in a 20 µL volume, containing 50 ng of genomic DNA, 1 × PCR buffer (Roche), 25 mM MgCl_2_, 10 mM dNTPs, 10 µM of each primer and 0.3 U of Taq DNA polymerase (Roche). The reactions were incubated at 95 °C for 5 min, followed by 35 cycles of 95 °C for 1 min, 61 °C for 1 min, 72 °C for 1 min 45 sec and a final step of 72 °C for 10 min. The quality of the PCR reaction was verified by electrophoresis on a 1.5% agarose gel in 1 × TAE. The PCR amplicons were purified with QIAquick PCR purification kit (Qiagen) and send to STAB VIDA (http://www.stabvida.com) to be sequenced with the primers listed in Supplementary Table S2, using the combinations established with the same number. The obtained sequences of the 20 varieties were aligned using the default alignment algorithm of EMBOSS Needle software (EMBL-EBI) and compared with the NCBI sequence for SNP identification (alignments are provided as Supplementary Figures S1 and S2). To achieve HRM varietal identification, primers surrounding the selected regions were designed (Supplementary Table S3).

PCR and HRM analysis was performed in a StepOne™ Real-Time PCR System (Applied Biosystems). All reactions were run in triplicates, in the presence of the negative (*O. europaea* L.) and blanc (NTC) controls. Reactions were performed for each primer set in a final volume of 20 µL containing the respective primer pair (5 µM of each primer), 20 ng of genomic DNA and the MeltDoctor™ HRM Master Mix (Thermo Fisher Scientific). The PCR amplification was followed by the HRM and included an initial denaturation step of 95 °C for 10 min followed by 40 cycles of 95 °C for 30 s, 58–60 °C for 30 s and 72 °C for 30 s, then a final extension step of 72 °C for 2 min. The melting curve was obtained in continuous, performed as follow: 95 °C for 30 s, 65 °C for 1 min rising 0.3 °C/s, 95 °C for 15 s. During the incremental melting step, fluorescence data was continuously acquired. A High-Resolution Melt Software v3.0.1 (Applied Biosystems) was used to analyse the data. After normalization and determining the temperature shift, the different melting curves of the several plots were generated.

### Purification of HRM products and Sanger sequencing analysis

To validate the results of HRM profiles, ten clones for each grapevine variety under study were analysed. Post-HRM products were purified using illustra ExoProStar 1-Step Kit (GE Healthcare Life Sciences) and the purified PCR products were sequenced in both directions, with the same PCR primers, using STAB VIDA services (http://www.stabvida.com). The CodonCode Aligner 4.0.4 (CodonCode Co., USA) was used to proofread, generate consensus sequences, and sequence alignment was performed with Geneious v5.6.4.

### Biosensor assay

The probes used in this assay were designed based on the SNPs detected in the *F3H* gene. Probe 25 was designed to target the SNPs located at the positions 1,039 and 1,040 bp. Target 11 is complementary to Probe 25 (Supplement Table S4). Targets 12 and 14 present a mismatch with Probe 25. Probe 35 was aimed to target the SNPs at three differential positions 1,039, 1,040 and 1,065 bp (Supplement Table S4). Target 15 is complementary to Probe 35; whereas Targets 16, 18 and 21 presented one, two and three base mismatches to Probe 35, respectively. A blastn (basic local alignment search tool nucleotide) using the NCBI genome database was performed to verify the specificity of the probes. All oligonucleotides were acquired from Frilabo (www.frilabo.pt) and used at a concentration of 0.25 µM.

Besides these oligonucleotides, leaf DNA from the grapevine varieties *Tinta Amarela* and *Malvasia Fina* were also tested. *Tinta Amarela* DNA sequence is complementary to both probes and *Malvasia Fina* DNA sequence is differentiated from *Tinta Amarela* in three and two SNPs targeted by Probe 25 and Probe 35, respectively. Two leaf DNA samples from two different species, *O. europaea* L. and *S. nigra* L., were used as negative controls.

Five different experiments were performed, testing different target sequences and DNA samples. The sequences were the following:

- Probe 25, Target 12, Target 14 and Target 11;
- Probe 25, Target 12, DNA from *Malvasia Fina* and DNA from *Tinta Amarela*;
- Probe 35, Target 16, Target 18, Target 21 and Target 15;
- Probe 35, Target 16, DNA from *Malvasia Fina* and DNA from *Tinta Amarela*.
- Probe 35, Target 18, DNA from *O. europaea* and DNA from *S. nigra* and Target 15.

A new Probe was designed *Tinta Roriz* specific, Probe 1065 (Supplementary Table S4), and was tested in the following sequence:

- Probe 1065, Target 17, DNA from *Malvasia Fina*, DNA from *Tinta Amarela* and, DNA from *Tinta Roriz*.

In order to study SNP position effect using Probe 35 a set of experiences were performed using targets with different SNP positions in relation to the Probe (Supplementary Table S4). Three tests were performed in the following sequence:

- Probe 35, Target SNP 3′ and Target 15.
- Probe 35, Target center and Target 15.
- Probe 35, Target SNP 5′ and Target 15.

Each experiment was performed at least three times.

During the experiments, the LPG was maintained at constant strain and temperature (37 °C). The optical data acquisition was performed using a fiber optic interrogation unit manufactured by HBM FiberSensing (www.fibersensing.com), model BraggMeter FS2200SA. This sensing system (considering this specific optical detection scheme and this specific LPG) has a detection limit of 62 ± 2 nM, a quantification limit of 209 ± 7 nM and a sensitivity of 78 ± 1 nM.RIU^19^.

The LPG surface was cleaned using a solution composed by ethanol 70% (v/v) and hydrochloric acid 1% (v/v) in a 1:1 ratio. Furthermore, Poly-L-Lysine (PLL) was used as a bilinker. In each cycle, the following sequence was executed: water, PLL, water, probe, water and DNA targets (with water between each target to assure that the differences observed were due to a chemical reaction and not just due to simple mass deposition effect). After each cycle, the LPG surface was cleaned using a diluted solution of nitric acid (HNO_3_ 1:3) to remove the different layers (PLL, Probe and complementary Target) attached to it. All solution injected in the chamber had the same volume (750 µL).

The LPG transmission spectra was recorded during 45 min with a 2-second interval for the PLL, Probe and DNA Target steps. When water was added, the data was recorded during 30 min with a 2-second interval. Each spectra is processed to determine the wavelength position of the LPG resonance. This wavelength is directly dependent of the refractive index of the fiber surface. A change in the refractive index, at a constant temperature (37 °C), reflects a chemical interaction between the biological materials on its surface. After data processing, the first twenty-five minutes of recordings were excluded (interval needed for each sample to establish thermal balance). The remaining data set is used to perform an arithmetic average and the resulting value is used as the measured resonance peak position for the protocol step. Finally, the average measured resonance peak of each step was subtracted to the average value of the initial water of the experience to calculate the wavelength shift obtained due to biochemical reactions. Simultaneously, a temperature sensor was positioned inside the reaction chamber near the LPG sensor and measurements were collected during all the experiments in order to adjust the resonance wavelength, once it is directly influenced by temperature, using a correction algorithmic developed previously for the system^19^.

In statistical analysis, values are given as mean ± SD as calculated from data from three independent experiences. The comparison between treatments were performed using shift values in comparison to the water reference. All treatments were compared to the probe values using a Student’s t-test or one-way ANOVA test to our endpoints data using the Statview software to test data’s statistical differences (P < 0.05).

### Accession numbers

Sequence data from this article can be found in the Gen-Bank database under the accession numbers: 2019324564; 2019324565; 2019324567; 2019324569; 2019324570; 2019324571; 2019324572; 2019324573; 2019324574; 2019324575.

## Electronic supplementary material

Supplementary information
