# Supplementary material for: Alternative SNP detection platforms, HRM and biosensors, for varietal identification in Vitis vinifera L. using F3H and LDOX genes
Source: Sci Rep. 2018 Apr 11;8:5850. doi: 10.1038/s41598-018-24158-9 (PMC5895793; doi:10.1038/s41598-018-24158-9)
Supplement: Supplementary file 1 — Supplementary information [file 41598_2018_24158_MOESM1_ESM.pdf]

Supplementary information

Alternative SNP detection platforms, HRM and biosensors, for varietal identification in *Vitis vinifera* L. using *F3H* and *LDOX* genes

Sónia Gomes[1,2,+]; Cláudia Castro[1,+]; Sara Barrias[1]; Leonor Pereira[1,2]; Pedro Jorge[3]; José R. Fernandes[1,3]; Paula Martins-Lopes[1,2,*]

[1]University of Trás-os-Montes and Alto Douro, P.O. Box 1013, 5000-911 Vila Real, Portugal.
[2]University of Lisboa, Faculty of Sciences, BioISI – Biosystems & Integrative Sciences Institute, Campo Grande, Lisboa, Portugal.
[3]INESC TEC, Rua do Campo Alegre n. 687, 4169-007 Porto, Portugal.
* plopes@utad.pt
+ these authors contributed equally to this work

Supplementary Table S1. List of 21 grapevine varieties used, corresponding code, berry colour and country of origin.

Grapevine variety	Code	Berry colour	Country of origin
Alicante Bouschet	AB	Red	France
Cabernet Sauvignon	CS	Red	France
Chardonnay	Ch	White	France
Donzelinho Tinto	DT	Red	Portugal
Fernão Pires	FP	White	Portugal
Gouveio	Gou	White	Portugal
Malvasia Fina	MF	White	Portugal
Merlot	M	Red	France
Moscatel Galego	MG	White	Portugal/ Spain
Rufete	Ruf	Red	Portugal
Sousão	Sou	Red	Portugal
Tinta Amarela	TA	Red	Portugal
Tinta Barroca	TB	Red	Portugal
Tinta Francisca	TFi	Red	Portugal
Tinta Roriz	TR	Red	Spain
Tinto Cão	TC	Red	Portugal
Touriga Brasileira	TBr	Red	Portugal
Touriga Franca	TF	Red	Portugal
Touriga Nacional	TN	Red	Portugal
Viosinho	Vio	White	Portugal

Supplementary Table S2. Characteristics of the primers used in the *F3H* and *LDOX* amplification and sequencing.

Primer Code	Sequence (5' → 3')	Length (bp)	T_m (°C)	GC%	Amplicon size (bp)
F_3H_4fwd	CGCAGCTGCTTGTTTTGAT	19	60.1	47.4	596
F_3H_4rev	TGAAAAATCAGGCCAAGACC	20	60.1	45.0	
F_3H_5fwd	ATGTCTGGTGGCAAGAAAGG	20	60.1	50.0	640
F_3H_5rev	AGGTTGAACGGTGATCCAAG	20	60.0	50.0	
F_3H_6fwd	TCAATTTCTACCCCCAATGC	20	59.8	45.0	786
F_3H_6rev	AAATCTGGTCAATGGGCTTG	20	59.9	45.0	
LDOX_1fwd	CAAGAGAGAAAGGGAAGG	18	54.9	50.0	500
LDOX_1rev	GGAAGATGAGGTGGAAGA	18	54.9	45.0	
LDOX_2fwd	GTATGCTAATGACCAGGC	18	54.0	50.0	490
LDOX_2rev	GGGGTAGTAGTTGATCTTC	18	55.0	50.0	
LDOX_3fwd	AAGGGAGACTAGAAAAGG	18	52.0	50.0	503
LDOX_3rev	GATGAAATACAACAAGGC	18	49.0	50.0	

Supplementary Table S3. Characteristics of the primers used in the F3H and LDOX HRM assays. Two specific HRM assays were designed for *F3H* gene with two specific regions. One amplicon with 375 bp (starting at 5 bp and ending at 379 bp); and another amplicon with 532 bp (starting at 975 bp and ending at 1506 bp). The HRM assay for *LDOX* gene was designed for a 450 bp region (starting at 386 bp and ending at 835 bp). The HRM assay validation as a technique for varietal discrimination was designed based on 201 bp specific region without any sequence variant in the 21 varieties.

Primer Code	Sequence (5' → 3')	Length (bp)	T_m (°C)	GC%	Amplicon size (bp)
F_3H_H1fwd	AGAGAAAGAAGGCGACGT	18	54.0	50.0	375
F_3H_H1rev	GATGGCTGGAAACGATGA	18	54.0	50.0	
F_3H_H2fwd	CTGTTGAAGGAGCTTTCG	18	54.0	50.0	532
F_3H_H2rev	GGCTTGGACTCTAACTTG	18	54.0	50.0	
LDOX_Hfwd	GCTTGCCAACAATGCTAG	18	54.0	50.0	450
LDOX_Hrev	GGACACTTGGGGTAGTAG	18	56.0	56.0	
LDOX_H2fwd	GCTTGCCAACAATGCTAG	19	54.0	50.0	201
LDOX_H2rev	GGACACTTGGGGTAGTAG	18	56.0	50.0	

Supplementary Table S4. List of oligonucleotides, probes and targets, used in the biosensor assay. In the sequence, marked in bold, are the SNPs positions.

Oligonucleotide code	Sequence (5' → 3')	Number of SNPs
**Probe 25**	C6-Aminolink- TCATAACCGGCGAAAGGCTGAAGCT	-
Target 11	AGCTTCAGCCTTTCGCCGGTTATGA	0
Target 12	AGCTTCAGCCTT**A**CGCCGGTTATGA	1
Target 14	AGCTTCAGCCT**AA**CGCCGGTTATGA	2
**Probe 35**	C6-Aminolink- GCGAAAGGCTGAAGCTAATCTTTTCTTTGTCTTTG	-
Target 15	CAAAGACAAAGAAAAGATTAGCTTCAGCCTTTCGC	0
Target 16	CAAAGACAAAGAAAAGATTAGCTTCAGCCTT**A**CGC	1
Target 17	CAAAGACAAAGAAAAGATTAGCTTCAGCCT**A**TCGC	1
Target 18	CAAAGACAAAGAAAAGATTAGCTTCAGCCT**AA**CGC	2
Target 20	CAAA**T**ACAAAGAAAAGATTAGCTTCAGCCTT**A**CGC	2
Target 21	CAAA**T**ACAAAGAAAAGATTAGCTTCAGCCT**AA**CGC	3
Target SNP3'	CAAAGACAAAGAAAAGATTAGCTTCAGCCTTTCG**G**	
Target SNP5'	**G**AAAGACAAAGAAAAGATTAGCTTCAGCCTTTCGC	
Target Medium	CAAAGACAAAGAAAA**C**ATTAGCTTCAGCCTTTCGC	
**Probe1065**	C6-Aminolink- GCGAAAGGCTGAAGCTAATCTTTTCTTTGT<u>A</u>TTTG	-

Supplementary Figure S1. The alignment of consensus *F3H* sequences of twenty *V. vinifera* varieties. The sequences were aligned using the default alignment algorithm of Geneious v5.6.4. The common forward and reverse primers flanked varieties specific regions of the twenty varieties considered under study. The target *F3H* amplified region was 4-1464 bp in length.

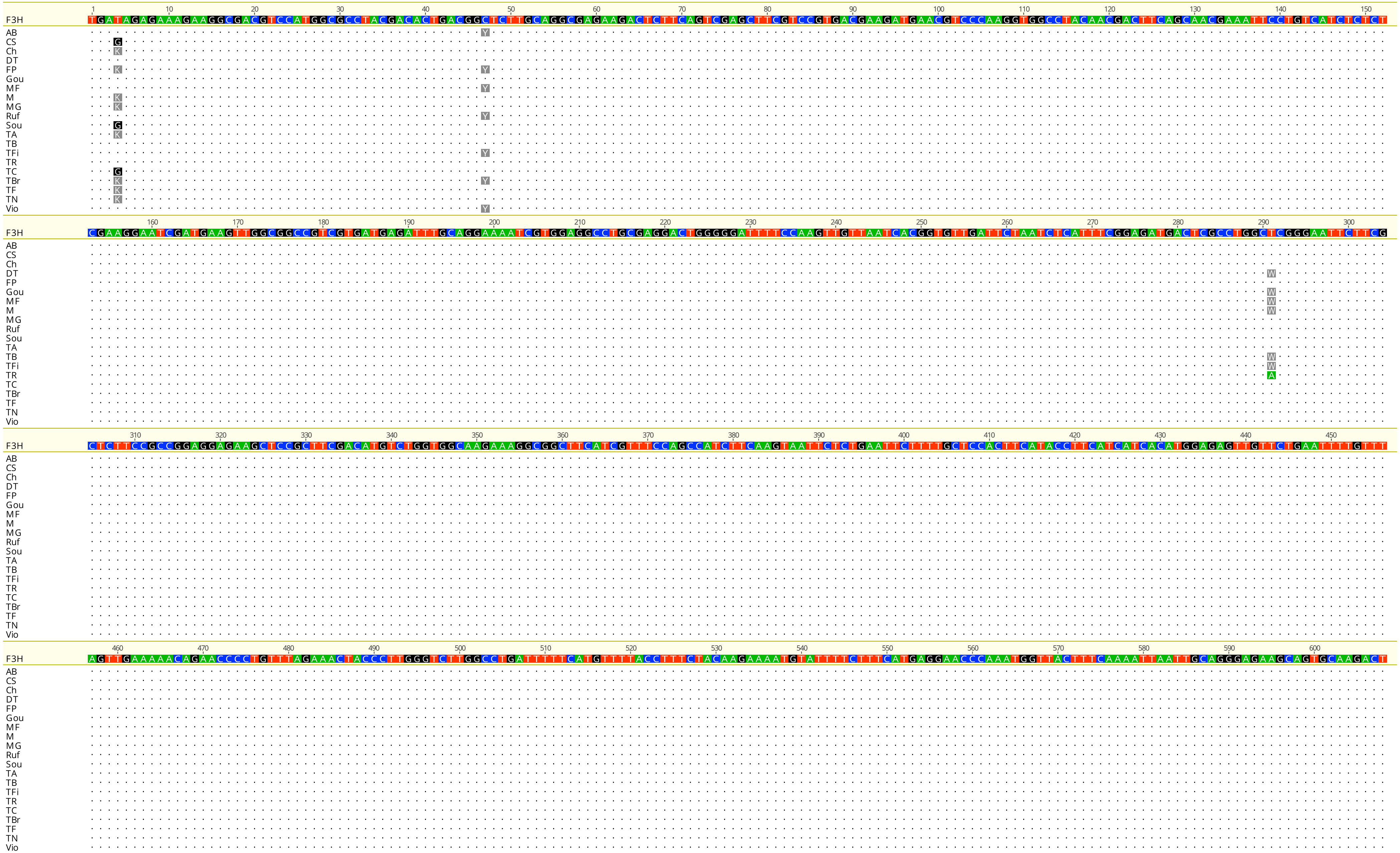
F3H
AB
CS
Ch
DT
FP
Gou
MF
M
MG
Ruf
Sou
TA
TB
TFi
TR
TC
TBr
TF
TN
Vio

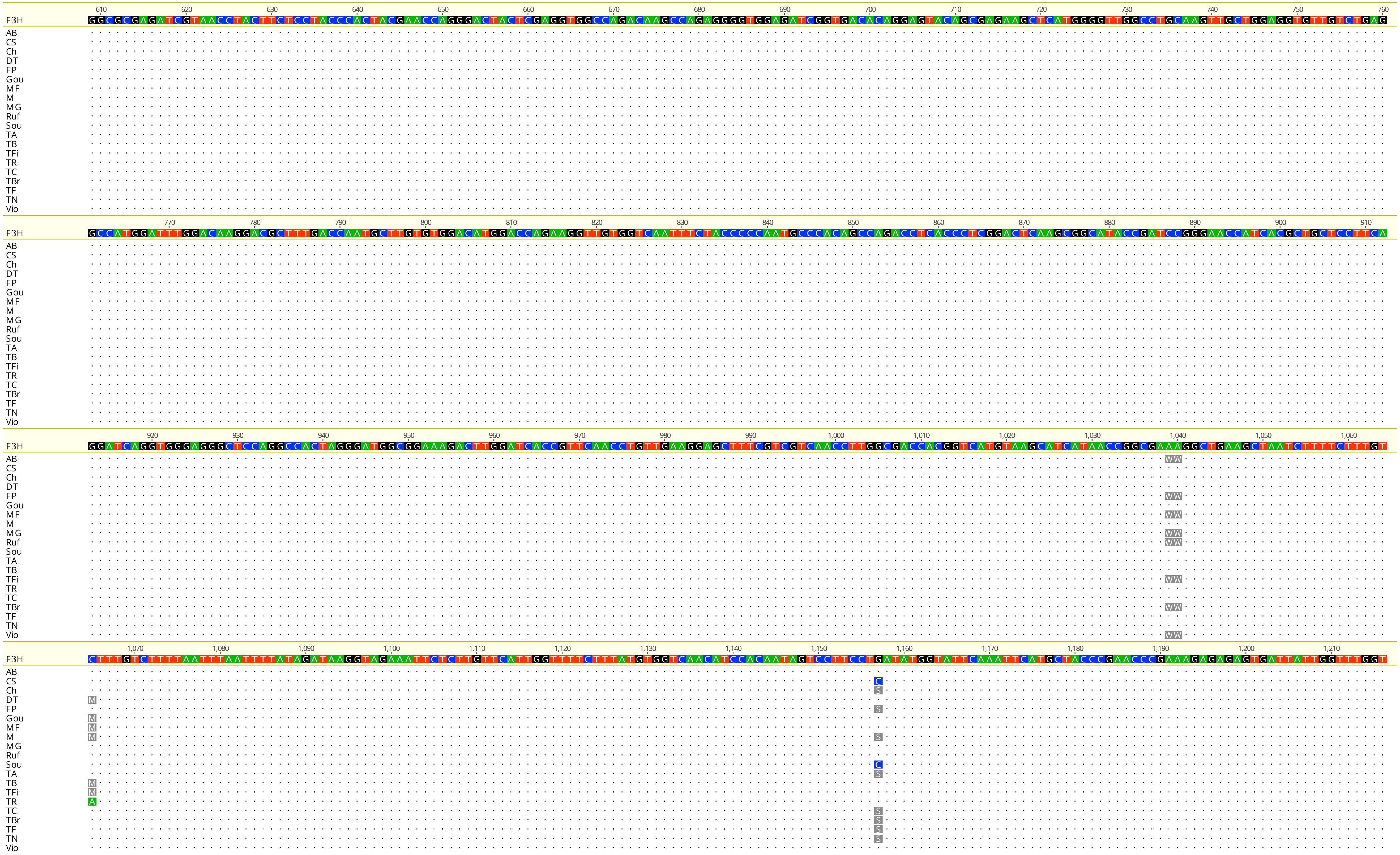
F3H
AB
CS
Ch
DT
FP
Gou
MF
M
MG
Ruf
Sou
TA
TB
TFi
TR
TC
TBr
TF
TN
Vio

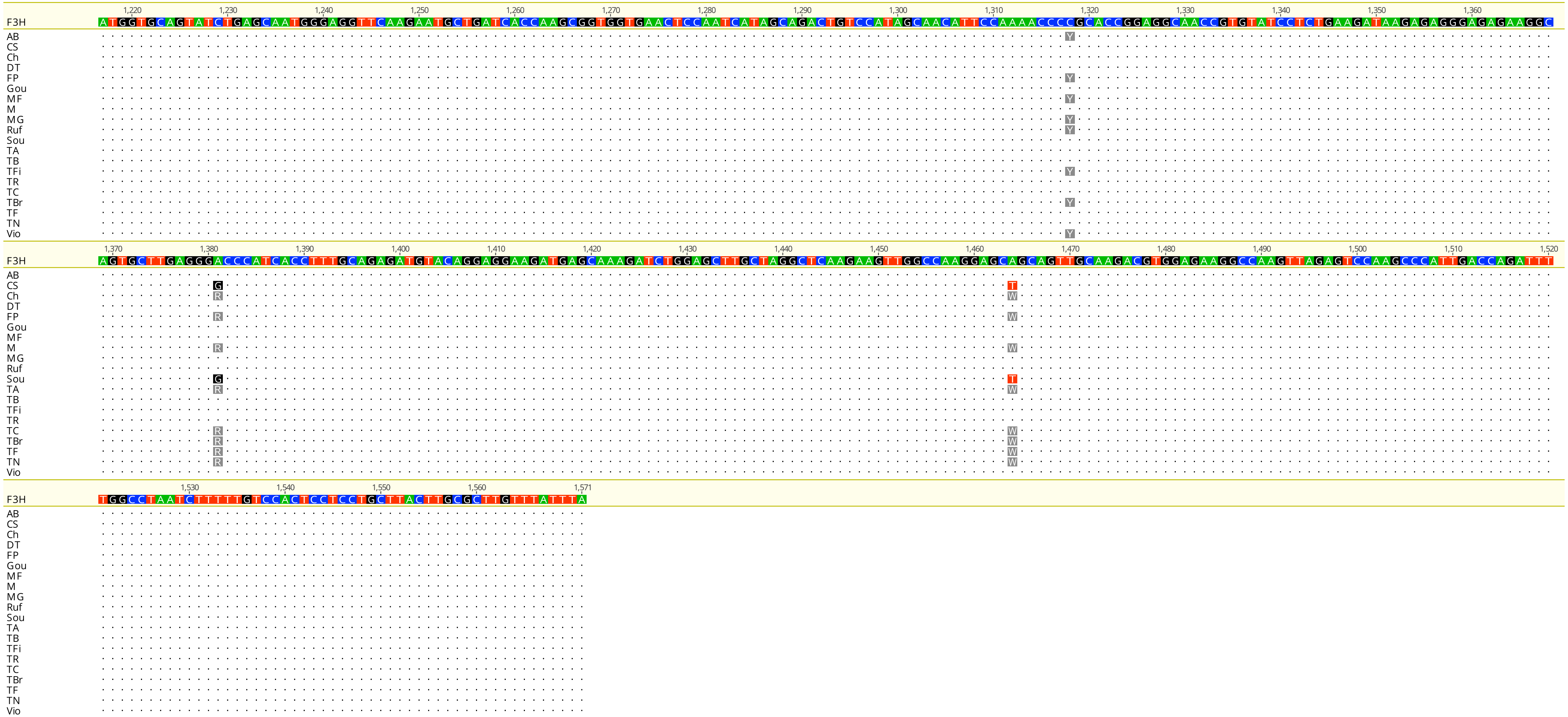
F3H
AB
CS
Ch
DT
FP
Gou
MF
M
MG
Ruf
Sou
TA
TB
TFi
TR
TC
TBr
TF
TN
Vio

Supplementary Figure S2. The alignment of consensus *LDOX* sequences of twenty *V. vinifera* varieties. The sequences were aligned using the default alignment algorithm of Geneious v5.6.4. The common forward and reverse primers flanked varieties specific regions, of the twenty varieties considered, under study. The target *LDOX* amplified region was 4-1192 bp in length.

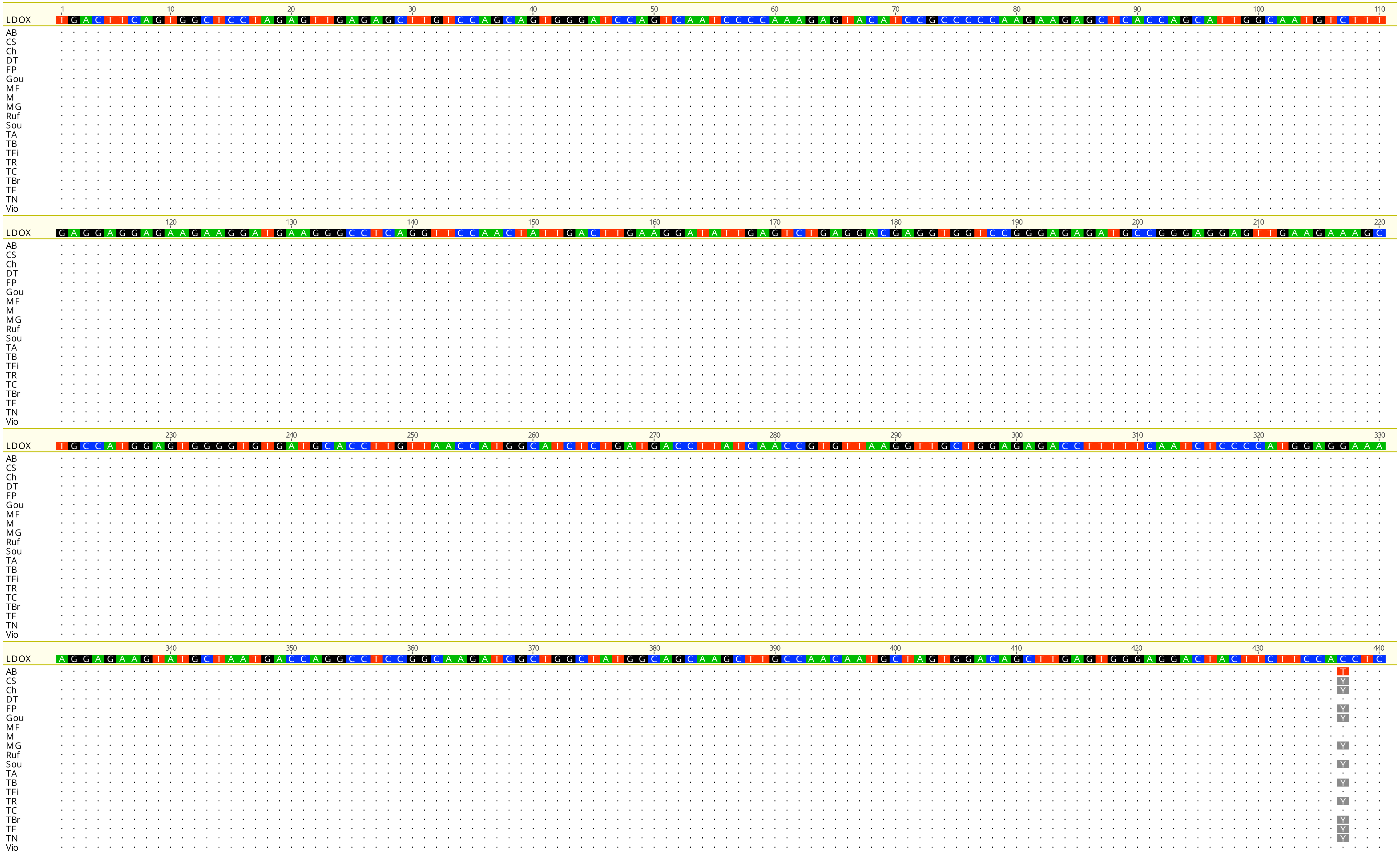
1 10 20 30 40 50 60 70 80 90 100 110
LDOX TGACTTCAGTGGCTCCTAGAGTTGAGAGCTTGTCCAGCAGTGGGATCCAGTCAATCCCCAAAGAGTACATCCGCCCCCAAGAAGAGCTCACCAGCATTGGCAATGTCTTT
120 130 140 150 160 170 180 190 200 210 220
LDOX GAGGAGGAGAAGAAGGATGAAGGGCCTCAGGTTCCAACTATTGACTTGAAGGATATTGAGTCTGAGGACGAGGTGGTCCGGGAGAGATGCCGGGAGGAGTTGAAGAAAGC
230 240 250 260 270 280 290 300 310 320 330
LDOX TGCCATGGAGTGGGGTGTGATGCACCTTGTTAACCATGGCATCTCTGATGACCTTATCAACCGTGTTAAGGTTGCTGGAGAGACCTTTTTCAATCTCCCCATGGAGGAAA
340 350 360 370 380 390 400 410 420 430 440
LDOX AGGAGAAGTATGCTAATGACCAGGCCTCCGGCAAGATCGCTGGCTATGGCAGCAAGCTTGCCAACAATGCTAGTGGACAGCTTGAGTGGGAGGACTACTTCTTCCACCTC
AB T
CS Y
Ch Y
DT
FP Y
Gou Y
MF
M
MG Y
Ruf
Sou Y
TA
TB Y
TFi
TR Y
TC
TBr Y
TF Y
TN Y
Vio

```
        450       460       470       480       490       500       510       520       530       540       550
LDOX    ATCTTCCCTGAAGACAAGCGCGATATGACCATCTGGCCTAAGACACCAAGCGACTACGTGTAAGTTCAATTCATTGTTTATGTAATTATACGATGTTTGCTGATCTGTTT
AB      ..............................................................................................................
CS      ..............................................................................................................
Ch      ..............................................................................................................
DT      ..............................................................................................................
FP      ..............................................................................................................
Gou     ..............................................................................................................
MF      ..............................................................................................................
M       ..............................................................................................................
MG      ..............................................................................................................
Ruf     ..............................................................................................................
Sou     ..............................................................................................................
TA      ..............................................................................................................
TB      ..............................................................................................................
TFi     ..............................................................................................................
TR      ..............................................................................................................
TC      ..............................................................................................................
TBr     ..............................................................................................................
TF      ..............................................................................................................
TN      ..............................................................................................................
Vio     ..............................................................................................................

        560       570       580       590       600       610       620       630       640       650       660
LDOX    TTCCTCTGGCCAAGTAGTTACTAACTGAATTTTCTATTATAAAAAAAAATCTGCAAATTTTACTAATTTTGAAATTGTTTCTGGGTTCTAAGAATTACTTGTTCAAACTT
AB      ..............................................................................................................
CS      ..............................................................................................................
Ch      ..............................................................................................................
DT      ..............................................................................................................
FP      ..............................................................................................................
Gou     ..............................................................................................................
MF      ..............................................................................................................
M       ..............................................................................................................
MG      ..............................................................................................................
Ruf     ..............................................................................................................
Sou     ..............................................................................................................
TA      ..............................................................................................................
TB      ..............................................................................................................
TFi     ..............................................................................................................
TR      ..............................................................................................................
TC      ..............................................................................................................
TBr     ..............................................................................................................
TF      ..............................................................................................................
TN      ..............................................................................................................
Vio     ..............................................................................................................

        670       680       690       700       710       720       730       740       750       760       770
LDOX    GTGATTGCAGTCCAGCAACCTGTGAGTACTCGGTGAAACTTAGGAGCCTGGCAACCAAGATACTATCGGTGCTATCGCTTGGGTTGGGATTGGAAGAAGGGAGACTAGAA
AB      ..............................................................................................................
CS      ..............................................................................................................
Ch      ..............................................................................................................
DT      ..............................................................................................................
FP      ..............................................................................................................
Gou     ..............................................................................................................
MF      ..............................................................................................................
M       ..............................................................................................................
MG      ..............................................................................................................
Ruf     ..............................................................................................................
Sou     ..............................................................................................................
TA      ..............................................................................................................
TB      ..............................................................................................................
TFi     ..............................................................................................................
TR      ..............................................................................................................
TC      ..............................................................................................................
TBr     ..............................................................................................................
TF      ..............................................................................................................
TN      ..............................................................................................................
Vio     ..............................................................................................................

        780       790       800       810       820       830       840       850       860       870       880
LDOX    AAGGAAGTTGGTGGGATGGAAGAGCTACTACTCCAAAAGAAGATCAACTACTACCCCAAGTGTCCCCAGCCTGAATTGGCTCTCGGGGTGGAAGCTCACACTGACGTGAG
AB      ..............................................................................................................
CS      ..............................................................................................................
Ch      ..............................................................................................................
DT      ..............................................................................................................
FP      ..............................................................................................................
Gou     ..............................................................................................................
MF      ..............................................................................................................
M       ..............................................................................................................
MG      ..............................................................................................................
Ruf     ..............................................................................................................
Sou     ..............................................................................................................
TA      ..............................................................................................................
TB      ..............................................................................................................
TFi     ..............................................................................................................
TR      ..............................................................................................................
TC      ..............................................................................................................
TBr     ..............................................................................................................
TF      ..............................................................................................................
TN      ..............................................................................................................
Vio     ..............................................................................................................
```

```
                890       900       910       920       930       940       950       960       970       980       990
LDOX  CGCTCTCACCTTCATCCTCCACAACATGGTACCCGGCCTGCAACTTTTCTATGAGGGCAAGTGGGTGACAGCCAAGTGTGTCCCCAACTCCATCATCATGCACATTGGAG
AB    ..............................................................................................................
CS    ..............................................................................................................
Ch    ..............................................................................................................
DT    ..............................................................................................................
FP    ..............................................................................................................
Gou   ..............................................................................................................
MF    ..............................................................................................................
M     ..............................................................................................................
MG    ..............................................................................................................
Ruf   ..............................................................................................................
Sou   ..............................................................................................................
TA    ..............................................................................................................
TB    ..............................................................................................................
TFi   ..............................................................................................................
TR    ..............................................................................................................
TC    ..............................................................................................................
TBr   ..............................................................................................................
TF    ..............................................................................................................
TN    ..............................................................................................................
Vio   ..............................................................................................................

               1,000     1,010     1,020     1,030     1,040     1,050     1,060     1,070     1,080     1,090     1,100
LDOX  ACACCATAGAGATTCTCAGCAATGGTAAGTACAAGAGTATTCTTCACAGGGGACTGGTCAACAAGGAGAAGGTGAGGATTTCATGGGCAGTTTTCTGCGAGCCGCCTAAG
AB    ..............................................................................................................
CS    ..............................................................................................................
Ch    ..............................................................................................................
DT    ..............................................................................................................
FP    ..............................................................................................................
Gou   ..............................................................................................................
MF    ..............................................................................................................
M     ..............................................................................................................
MG    ..............................................................................................................
Ruf   ..............................................................................................................
Sou   ..............................................................................................................
TA    ..............................................................................................................
TB    ..............................................................................................................
TFi   ..............................................................................................................
TR    ..............................................................................................................
TC    ..............................................................................................................
TBr   ..............................................................................................................
TF    ..............................................................................................................
TN    ..............................................................................................................
Vio   ..............................................................................................................

               1,110     1,120     1,130     1,140     1,150     1,160     1,170     1,180     1,190     1,200     1,210
LDOX  GAGAAGATCATCCTGAAGCCACTGCCAGAGACGGTGTCTGAGACTGAGCCACCACTCTTCCCACCTCGCACCTTTTCCCAACATATTCAGCACAAGCTCTTCAGGAAGAC
AB    ..............................................................................................................
CS    ..............................................................................................................
Ch    ..............................................................................................................
DT    ..............................................................................................................
FP    ..............................................................................................................
Gou   ..............................................................................................................
MF    ..............................................................................................................
M     ..............................................................................................................
MG    ..............................................................................................................
Ruf   ..............................................................................................................
Sou   ..............................................................................................................
TA    ..............................................................................................................
TB    ..............................................................................................................
TFi   ..............................................................................................................
TR    ..............................................................................................................
TC    ..............................................................................................................
TBr   ..............................................................................................................
TF    ..............................................................................................................
TN    ..............................................................................................................
Vio   ..............................................................................................................

               1,220          1,235
LDOX  CCAGGAGGCTCTACTCTCCAAATGA
AB    .........................
CS    .........................
Ch    .........................
DT    .........................
FP    .........................
Gou   .........................
MF    .........................
M     .........................
MG    .........................
Ruf   .........................
Sou   .........................
TA    .........................
TB    .........................
TFi   .........................
TR    .........................
TC    .........................
TBr   .........................
TF    .........................
TN    .........................
Vio   .........................
```
